# Tetra-*tert*-butyl 13,14-dioxapenta­cyclo­[8.2.1.1^4,7^.0^2,9^.0^3,8^]tetra­deca-5,11-diene-5,6,11,12-tetra­carboxyl­ate

**DOI:** 10.1107/S1600536812039220

**Published:** 2012-09-22

**Authors:** Alan J. Lough, Kelsey Jack, William Tam

**Affiliations:** a Department of Chemistry, University of Toronto, Toronto, Ontario, Canada M5S 3H6; b Department of Chemistry, University of Guelph, Guelph, Ontario, Canada N1G 2W1

## Abstract

The stereochemistry of the title compound, C_32_H_44_O_10_, at the cyclo­butane ring is *cis-anti-cis*. The mol­ecule lies across an inversion center. In the crystal, weak C—H⋯O hydrogen bonds connect mol­ecules into chains along [100], forming *R*_2_^2^(6) rings.

## Related literature

For related structures, see: Lough *et al.* (2012*a* ▶,*b* ▶). For the synthetic background, see: Ballantine *et al.* (2009 ▶). For hydrogen-bond motifs, see: Bernstein *et al.* (1995 ▶).
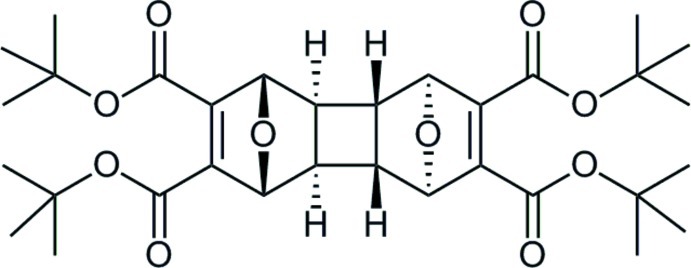


## Experimental

### 

#### Crystal data

C_32_H_44_O_10_
*M* *_r_* = 588.67
Triclinic, 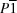

*a* = 5.8376 (10) Å
*b* = 9.4895 (17) Å
*c* = 14.924 (3) Å
α = 99.926 (4)°
β = 98.545 (4)°
γ = 100.462 (4)°
*V* = 786.9 (2) Å^3^
*Z* = 1
Mo *K*α radiation
μ = 0.09 mm^−1^
*T* = 150 K
0.45 × 0.25 × 0.15 mm

#### Data collection

Bruker Kappa APEX DUO CCD diffractometer
Absorption correction: multi-scan (*SADABS*; Bruker, 2007 ▶) *T*_min_ = 0.698, *T*_max_ = 0.746
6490 measured reflections
3559 independent reflections
3024 reflections with *I* > 2σ(*I*)
*R*_int_ = 0.015

#### Refinement

*R*[*F* ^2^ > 2σ(*F* ^2^)] = 0.040
*wR*(*F* ^2^) = 0.104
*S* = 1.04
3559 reflections
196 parameters
H-atom parameters constrained
Δρ_max_ = 0.33 e Å^−3^
Δρ_min_ = −0.21 e Å^−3^

### 

Data collection: *APEX2* (Bruker, 2007 ▶); cell refinement: *SAINT* (Bruker, 2007 ▶); data reduction: *SAINT*; program(s) used to solve structure: *SHELXS97* (Sheldrick, 2008 ▶); program(s) used to refine structure: *SHELXL97* (Sheldrick, 2008 ▶); molecular graphics: *PLATON* (Spek, 2009 ▶); software used to prepare material for publication: *SHELXTL* (Sheldrick, 2008 ▶).

## Supplementary Material

Crystal structure: contains datablock(s) global, I. DOI: 10.1107/S1600536812039220/hb6953sup1.cif

Structure factors: contains datablock(s) I. DOI: 10.1107/S1600536812039220/hb6953Isup2.hkl

Supplementary material file. DOI: 10.1107/S1600536812039220/hb6953Isup3.cml

Additional supplementary materials:  crystallographic information; 3D view; checkCIF report

## Figures and Tables

**Table 1:** Hydrogen-bond geometry (Å, °)

*D*—H⋯*A*	*D*—H	H⋯*A*	*D*⋯*A*	*D*—H⋯*A*
C2—H2*A*⋯O1^i^	1.00	2.47	3.2118 (16)	130

Symmetry code: (i) 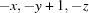.

**Fig. 1.**
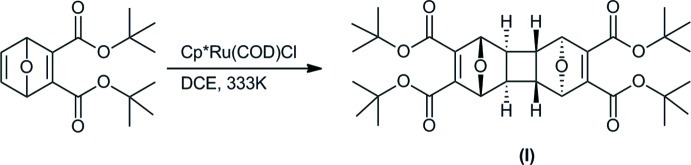
Reaction scheme

**Fig. 2.**
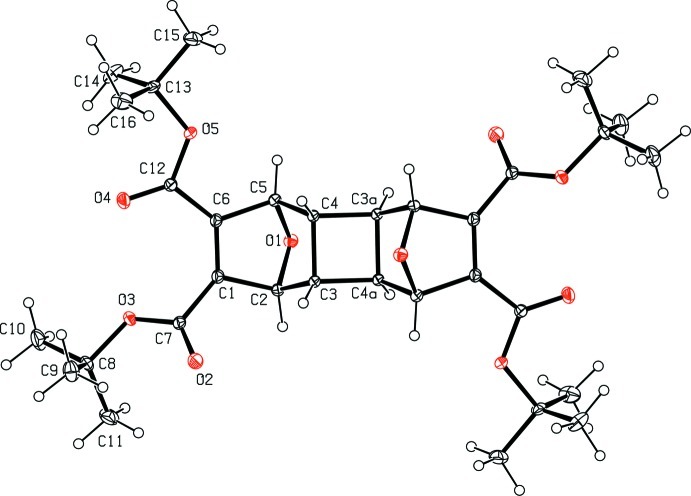
The molecular structure of (I) showing 30% probability ellipsoids (symmetry code (a): -x + 1, -y + 1, -z).

**Fig. 3.**
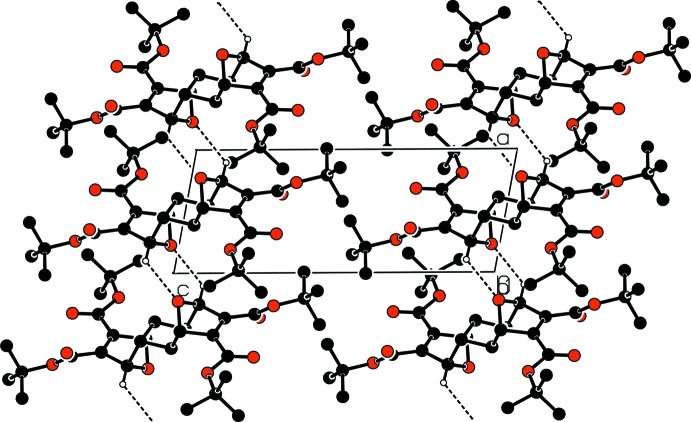
Part of the crystal structure showing weak hydrogen bonds as dashed lines.

## supplementary crystallographic information

### Comment

We have recently investigated the Ru-catalyzed isomerization and dimerization reaction of oxanorbornadiene compounds (Ballantine *et al.*, 2009). When dissolved in 1,2-dichloroethane in the presence of Cp*Ru(COD)Cl, *tert*-butoxy-7-oxabicyclo[2,2,1]hepta-2,5-diene-2,3-dicarboxylate will dimerize into the title compound (I) (see Fig. 1). The desired product was resolved using fractional crystallization in hexanes. The stereochemistry of the product was determined by this single-crystal X-ray analysis and was found to have a *cis*-anti-*cis* stereochemistry at the cyclobutane ring of the dimer.

The molecular structure of (I) is shown in Fig. 2. In the crystal, weak C—H···O hydrogen bonds connect molecules into one-dimensional chains (Fig. 3) along [100] forming *R*^2^_2_(6) rings (Bernstein *et al.*, 1995).

For related structures see the preceding (Lough *et al.*, 2012*a*) and following (Lough *et al.*, 2012*b*) papers

### Experimental

*tert*-Butoxy-7-oxabicyclo[2,2,1]hepta-2,5-diene-2,3-dicarboxylate 1 (45 mg, 0.15 mmol) was weighed into an oven-dried vial, purged with nitrogen and transferred into a Dry Box. In the Dry Box, Cp*Ru(COD)Cl (10 mol%) was added to another oven dried vial and dissolved in 1,2-dichloroethane (0.5 ml). The Ru-catalyst was then transferred into the vial containing the 7-oxanorbornadiene. The vial was sealed with a screw cap and removed from the Dry Box. The reaction was heated at 333 K with stirring for 20 h. The crude product was purified by column chromatography (EtOAc:hexanes = 2:3) followed by recrystallization in hexanes to give the dimer (I). Colourless blocks were grown by slow evaportation of a solution of (I) in hexanes.

### Refinement

Hydrogen atoms were placed in calculated positions with C—H distances of 0.98 and 1.00 Å. They were included in the refinement in a riding-model approximation with *U*_iso_(H) = 1.2*U*_eq_(C) or 1.5*U*_eq_(C_methyl_).

### Crystal data

**Table:**

C_32_H_44_O_10_	*Z* = 1
*M**_r_* = 588.67	*F*(000) = 316
Triclinic, *P*1	*D*_x_ = 1.242 Mg m^−^^3^
Hall symbol: -P 1	Mo *K*α radiation, λ = 0.71073 Å
*a* = 5.8376 (10) Å	Cell parameters from 4249 reflections
*b* = 9.4895 (17) Å	θ = 2.2–27.5°
*c* = 14.924 (3) Å	µ = 0.09 mm^−^^1^
α = 99.926 (4)°	*T* = 150 K
β = 98.545 (4)°	Block, colourless
γ = 100.462 (4)°	0.45 × 0.25 × 0.15 mm
*V* = 786.9 (2) Å^3^	

### Data collection

**Table:**

Bruker Kappa APEX DUO CCD diffractometer	3559 independent reflections
Radiation source: fine-focus sealed tube	3024 reflections with *I* > 2σ(*I*)
Bruker Triumph monochromator	*R*_int_ = 0.015
φ and ω scans	θ_max_ = 27.5°, θ_min_ = 1.4°
Absorption correction: multi-scan (*SADABS*; Bruker, 2007)	*h* = −7→4
*T*_min_ = 0.698, *T*_max_ = 0.746	*k* = −12→12
6490 measured reflections	*l* = −19→18

### Refinement

**Table:**

Refinement on *F*^2^	Primary atom site location: structure-invariant direct methods
Least-squares matrix: full	Secondary atom site location: difference Fourier map
*R*[*F*^2^ > 2σ(*F*^2^)] = 0.040	Hydrogen site location: inferred from neighbouring sites
*wR*(*F*^2^) = 0.104	H-atom parameters constrained
*S* = 1.04	*w* = 1/[σ^2^(*F*_o_^2^) + (0.0475*P*)^2^ + 0.3125*P*] where *P* = (*F*_o_^2^ + 2*F*_c_^2^)/3
3559 reflections	(Δ/σ)_max_ = 0.001
196 parameters	Δρ_max_ = 0.33 e Å^−^^3^
0 restraints	Δρ_min_ = −0.21 e Å^−^^3^

### Special details

**Table:**

Geometry. All e.s.d.'s (except the e.s.d. in the dihedral angle between two l.s. planes) are estimated using the full covariance matrix. The cell e.s.d.'s are taken into account individually in the estimation of e.s.d.'s in distances, angles and torsion angles; correlations between e.s.d.'s in cell parameters are only used when they are defined by crystal symmetry. An approximate (isotropic) treatment of cell e.s.d.'s is used for estimating e.s.d.'s involving l.s. planes.
Refinement. Refinement of *F*^2^ against ALL reflections. The weighted *R*-factor *wR* and goodness of fit *S* are based on *F*^2^, conventional *R*-factors *R* are based on *F*, with *F* set to zero for negative *F*^2^. The threshold expression of *F*^2^ > σ(*F*^2^) is used only for calculating *R*-factors(gt) *etc*. and is not relevant to the choice of reflections for refinement. *R*-factors based on *F*^2^ are statistically about twice as large as those based on *F*, and *R*- factors based on ALL data will be even larger.

### Fractional atomic coordinates and isotropic or equivalent isotropic displacement parameters (Å^2^)

**Table:**

	*x*	*y*	*z*	*U*_iso_*/*U*_eq_	
O1	0.22773 (14)	0.65121 (9)	0.02699 (6)	0.01803 (19)	
O2	0.35940 (19)	0.52069 (10)	0.29004 (7)	0.0282 (2)	
O3	0.26592 (16)	0.74251 (10)	0.32725 (6)	0.0217 (2)	
O4	0.68115 (18)	0.95566 (11)	0.29352 (7)	0.0298 (2)	
O5	0.80759 (16)	0.97519 (9)	0.15871 (6)	0.0207 (2)	
C1	0.3636 (2)	0.67330 (13)	0.18207 (8)	0.0174 (2)	
C2	0.2523 (2)	0.55902 (13)	0.09395 (8)	0.0165 (2)	
H2A	0.1029	0.4910	0.0973	0.020*	
C3	0.4520 (2)	0.48426 (13)	0.06656 (8)	0.0154 (2)	
H3	0.5361	0.4416	0.1152	0.018*	
C4	0.6083 (2)	0.61021 (12)	0.03288 (8)	0.0146 (2)	
H4	0.7801	0.6364	0.0622	0.018*	
C5	0.4701 (2)	0.73280 (13)	0.05005 (8)	0.0164 (2)	
H5A	0.5070	0.8137	0.0160	0.020*	
C6	0.5012 (2)	0.78123 (13)	0.15483 (8)	0.0167 (2)	
C7	0.3337 (2)	0.63815 (13)	0.27330 (8)	0.0180 (2)	
C8	0.2067 (2)	0.72280 (14)	0.41859 (8)	0.0211 (3)	
C9	0.4200 (3)	0.69922 (18)	0.48118 (10)	0.0332 (3)	
H9A	0.5579	0.7767	0.4830	0.050*	
H9B	0.4535	0.6038	0.4572	0.050*	
H9C	0.3865	0.7020	0.5438	0.050*	
C10	0.1460 (3)	0.86826 (18)	0.45469 (11)	0.0375 (4)	
H10A	0.0047	0.8794	0.4143	0.056*	
H10B	0.2793	0.9481	0.4555	0.056*	
H10C	0.1146	0.8712	0.5176	0.056*	
C11	−0.0074 (3)	0.59823 (18)	0.40294 (11)	0.0347 (3)	
H11A	−0.1297	0.6103	0.3540	0.052*	
H11B	−0.0703	0.5989	0.4603	0.052*	
H11C	0.0392	0.5049	0.3844	0.052*	
C12	0.6722 (2)	0.91271 (13)	0.21227 (8)	0.0169 (2)	
C13	0.9780 (2)	1.11711 (14)	0.19571 (9)	0.0212 (3)	
C14	0.8388 (3)	1.23483 (16)	0.21650 (13)	0.0371 (4)	
H14A	0.7595	1.2190	0.2685	0.056*	
H14B	0.7199	1.2307	0.1618	0.056*	
H14C	0.9469	1.3311	0.2327	0.056*	
C15	1.1022 (3)	1.13826 (18)	0.11508 (11)	0.0380 (4)	
H15A	0.9856	1.1420	0.0615	0.057*	
H15B	1.1795	1.0563	0.0992	0.057*	
H15C	1.2216	1.2301	0.1325	0.057*	
C16	1.1550 (3)	1.10905 (18)	0.27928 (11)	0.0344 (4)	
H16A	1.0756	1.1054	0.3325	0.052*	
H16B	1.2846	1.1957	0.2937	0.052*	
H16C	1.2190	1.0208	0.2655	0.052*	

### Atomic displacement parameters (Å^2^)

**Table:**

	*U*^11^	*U*^22^	*U*^33^	*U*^12^	*U*^13^	*U*^23^
O1	0.0147 (4)	0.0190 (4)	0.0187 (4)	0.0016 (3)	0.0010 (3)	0.0033 (3)
O2	0.0417 (6)	0.0206 (5)	0.0259 (5)	0.0099 (4)	0.0126 (4)	0.0059 (4)
O3	0.0300 (5)	0.0195 (4)	0.0184 (4)	0.0067 (4)	0.0118 (4)	0.0039 (3)
O4	0.0303 (5)	0.0322 (6)	0.0183 (5)	−0.0081 (4)	0.0071 (4)	−0.0046 (4)
O5	0.0246 (5)	0.0151 (4)	0.0186 (4)	−0.0039 (3)	0.0061 (3)	0.0003 (3)
C1	0.0169 (6)	0.0167 (6)	0.0176 (6)	0.0030 (4)	0.0048 (4)	0.0004 (4)
C2	0.0154 (6)	0.0167 (6)	0.0158 (6)	−0.0001 (4)	0.0041 (4)	0.0018 (4)
C3	0.0157 (6)	0.0141 (5)	0.0145 (6)	−0.0005 (4)	0.0035 (4)	0.0009 (4)
C4	0.0137 (5)	0.0144 (6)	0.0139 (5)	−0.0002 (4)	0.0029 (4)	0.0009 (4)
C5	0.0160 (6)	0.0155 (6)	0.0160 (6)	0.0004 (4)	0.0025 (4)	0.0017 (4)
C6	0.0169 (6)	0.0159 (6)	0.0166 (6)	0.0036 (4)	0.0045 (4)	0.0005 (4)
C7	0.0159 (6)	0.0175 (6)	0.0181 (6)	−0.0006 (4)	0.0046 (4)	0.0000 (4)
C8	0.0267 (7)	0.0247 (7)	0.0144 (6)	0.0071 (5)	0.0088 (5)	0.0045 (5)
C9	0.0363 (8)	0.0394 (8)	0.0230 (7)	0.0129 (6)	0.0008 (6)	0.0028 (6)
C10	0.0595 (11)	0.0365 (9)	0.0257 (8)	0.0253 (8)	0.0185 (7)	0.0062 (6)
C11	0.0293 (8)	0.0438 (9)	0.0320 (8)	0.0004 (6)	0.0146 (6)	0.0107 (7)
C12	0.0168 (6)	0.0155 (6)	0.0179 (6)	0.0024 (4)	0.0043 (4)	0.0022 (4)
C13	0.0200 (6)	0.0161 (6)	0.0227 (6)	−0.0052 (5)	0.0013 (5)	0.0031 (5)
C14	0.0375 (9)	0.0171 (7)	0.0513 (10)	0.0022 (6)	0.0024 (7)	0.0016 (6)
C15	0.0415 (9)	0.0357 (8)	0.0305 (8)	−0.0128 (7)	0.0109 (6)	0.0080 (6)
C16	0.0213 (7)	0.0430 (9)	0.0339 (8)	−0.0044 (6)	−0.0031 (6)	0.0137 (7)

### Geometric parameters (Å, º)

**Table:**

O1—C5	1.4442 (14)	C8—C10	1.5164 (19)
O1—C2	1.4453 (14)	C8—C9	1.5171 (19)
O2—C7	1.2135 (16)	C9—H9A	0.9800
O3—C7	1.3220 (15)	C9—H9B	0.9800
O3—C8	1.4892 (15)	C9—H9C	0.9800
O4—C12	1.2010 (16)	C10—H10A	0.9800
O5—C12	1.3402 (15)	C10—H10B	0.9800
O5—C13	1.4840 (14)	C10—H10C	0.9800
C1—C6	1.3421 (17)	C11—H11A	0.9800
C1—C7	1.4839 (17)	C11—H11B	0.9800
C1—C2	1.5253 (16)	C11—H11C	0.9800
C2—C3	1.5401 (16)	C13—C14	1.513 (2)
C2—H2A	1.0000	C13—C15	1.518 (2)
C3—C4^i^	1.5498 (16)	C13—C16	1.5190 (18)
C3—C4	1.5686 (16)	C14—H14A	0.9800
C3—H3	1.0000	C14—H14B	0.9800
C4—C5	1.5392 (16)	C14—H14C	0.9800
C4—C3^i^	1.5498 (16)	C15—H15A	0.9800
C4—H4	1.0000	C15—H15B	0.9800
C5—C6	1.5255 (16)	C15—H15C	0.9800
C5—H5A	1.0000	C16—H16A	0.9800
C6—C12	1.4876 (16)	C16—H16B	0.9800
C8—C11	1.515 (2)	C16—H16C	0.9800
			
C5—O1—C2	96.18 (8)	H9A—C9—H9B	109.5
C7—O3—C8	120.96 (10)	C8—C9—H9C	109.5
C12—O5—C13	121.03 (10)	H9A—C9—H9C	109.5
C6—C1—C7	134.31 (11)	H9B—C9—H9C	109.5
C6—C1—C2	105.44 (10)	C8—C10—H10A	109.5
C7—C1—C2	119.46 (10)	C8—C10—H10B	109.5
O1—C2—C1	101.00 (9)	H10A—C10—H10B	109.5
O1—C2—C3	102.44 (9)	C8—C10—H10C	109.5
C1—C2—C3	105.96 (9)	H10A—C10—H10C	109.5
O1—C2—H2A	115.2	H10B—C10—H10C	109.5
C1—C2—H2A	115.2	C8—C11—H11A	109.5
C3—C2—H2A	115.2	C8—C11—H11B	109.5
C2—C3—C4^i^	113.90 (9)	H11A—C11—H11B	109.5
C2—C3—C4	100.76 (9)	C8—C11—H11C	109.5
C4^i^—C3—C4	90.45 (9)	H11A—C11—H11C	109.5
C2—C3—H3	116.0	H11B—C11—H11C	109.5
C4^i^—C3—H3	116.0	O4—C12—O5	126.38 (11)
C4—C3—H3	116.0	O4—C12—C6	124.31 (11)
C5—C4—C3^i^	115.39 (9)	O5—C12—C6	109.30 (10)
C5—C4—C3	101.01 (9)	O5—C13—C14	108.34 (11)
C3^i^—C4—C3	89.55 (9)	O5—C13—C15	102.62 (10)
C5—C4—H4	115.7	C14—C13—C15	110.74 (13)
C3^i^—C4—H4	115.7	O5—C13—C16	111.42 (11)
C3—C4—H4	115.7	C14—C13—C16	112.83 (13)
O1—C5—C6	101.33 (9)	C15—C13—C16	110.40 (12)
O1—C5—C4	101.79 (9)	C13—C14—H14A	109.5
C6—C5—C4	106.44 (9)	C13—C14—H14B	109.5
O1—C5—H5A	115.2	H14A—C14—H14B	109.5
C6—C5—H5A	115.2	C13—C14—H14C	109.5
C4—C5—H5A	115.2	H14A—C14—H14C	109.5
C1—C6—C12	129.04 (11)	H14B—C14—H14C	109.5
C1—C6—C5	105.29 (10)	C13—C15—H15A	109.5
C12—C6—C5	125.30 (11)	C13—C15—H15B	109.5
O2—C7—O3	126.77 (12)	H15A—C15—H15B	109.5
O2—C7—C1	120.41 (11)	C13—C15—H15C	109.5
O3—C7—C1	112.71 (10)	H15A—C15—H15C	109.5
O3—C8—C11	108.87 (10)	H15B—C15—H15C	109.5
O3—C8—C10	101.91 (10)	C13—C16—H16A	109.5
C11—C8—C10	111.03 (12)	C13—C16—H16B	109.5
O3—C8—C9	110.50 (11)	H16A—C16—H16B	109.5
C11—C8—C9	112.93 (12)	C13—C16—H16C	109.5
C10—C8—C9	111.03 (12)	H16A—C16—H16C	109.5
C8—C9—H9A	109.5	H16B—C16—H16C	109.5
C8—C9—H9B	109.5		
			
C5—O1—C2—C1	51.53 (10)	C2—C1—C6—C5	0.72 (12)
C5—O1—C2—C3	−57.73 (10)	O1—C5—C6—C1	32.18 (12)
C6—C1—C2—O1	−33.35 (12)	C4—C5—C6—C1	−73.86 (12)
C7—C1—C2—O1	155.41 (10)	O1—C5—C6—C12	−154.29 (11)
C6—C1—C2—C3	73.14 (12)	C4—C5—C6—C12	99.66 (13)
C7—C1—C2—C3	−98.11 (12)	C8—O3—C7—O2	−1.27 (19)
O1—C2—C3—C4^i^	−61.22 (11)	C8—O3—C7—C1	174.85 (10)
C1—C2—C3—C4^i^	−166.66 (9)	C6—C1—C7—O2	−123.53 (16)
O1—C2—C3—C4	34.11 (10)	C2—C1—C7—O2	44.64 (17)
C1—C2—C3—C4	−71.33 (11)	C6—C1—C7—O3	60.08 (18)
C2—C3—C4—C5	1.36 (10)	C2—C1—C7—O3	−131.75 (11)
C4^i^—C3—C4—C5	115.81 (10)	C7—O3—C8—C11	−63.78 (15)
C2—C3—C4—C3^i^	−114.45 (10)	C7—O3—C8—C10	178.84 (12)
C4^i^—C3—C4—C3^i^	0.0	C7—O3—C8—C9	60.78 (15)
C2—O1—C5—C6	−51.21 (10)	C13—O5—C12—O4	−4.86 (19)
C2—O1—C5—C4	58.47 (10)	C13—O5—C12—C6	173.80 (10)
C3^i^—C4—C5—O1	58.36 (12)	C1—C6—C12—O4	−14.3 (2)
C3—C4—C5—O1	−36.43 (10)	C5—C6—C12—O4	173.77 (12)
C3^i^—C4—C5—C6	164.08 (10)	C1—C6—C12—O5	167.03 (12)
C3—C4—C5—C6	69.28 (11)	C5—C6—C12—O5	−4.92 (16)
C7—C1—C6—C12	−3.2 (2)	C12—O5—C13—C14	−67.15 (15)
C2—C1—C6—C12	−172.48 (11)	C12—O5—C13—C15	175.69 (12)
C7—C1—C6—C5	170.05 (13)	C12—O5—C13—C16	57.55 (16)

Symmetry code: (i) −*x*+1, −*y*+1, −*z*.

### Hydrogen-bond geometry (Å, º)

**Table:**

*D*—H···*A*	*D*—H	H···*A*	*D*···*A*	*D*—H···*A*
C2—H2*A*···O1^ii^	1.00	2.47	3.2118 (16)	130

Symmetry code: (ii) −*x*, −*y*+1, −*z*.
